# Design and Characterization of a Bioinspired Polyvinyl Alcohol Matrix with Structural Foam-Wall Microarchitectures for Potential Tissue Engineering Applications

**DOI:** 10.3390/polym14081585

**Published:** 2022-04-13

**Authors:** Ching-Cheng Huang

**Affiliations:** 1 Department of Biomedical Engineering, Ming-Chuan University, Guishan District, Taoyuan 320-33, Taiwan; junas.tw@yahoo.com.tw; 2 PARSD Biomedical Material Research Center, Xitun District, Taichung 407-49, Taiwan

**Keywords:** polyvinyl alcohol, soft matrix, composite matrix, human hepatoblastoma, bioinspired design

## Abstract

Traditional medical soft matrix used in a surgical treatment or in wound management was not good enough in both the structural support and interconnectivity to be applied in tissue engineering as a scaffold. Avian skeleton and feather rachises might be good reference objects to mimic in designing a scaffold material with good structural support and high interconnectivity because of its structural foam-wall microarchitectures and structural pneumaticity. In this study, a biomimetic airstream pore-foaming process was built up and the corresponding new medical soft matrix derived from polyvinyl alcohol matrix (PVAM) with air cavities inspired by avian skeleton and feather rachises was prepared. Furthermore, the resulting medical soft matrix and bovine Achilles tendon type I collagen could be employed to prepare a new collagen-containing composite matrix. Characterization, thermal stability and cell morphology of the bioinspired PVA matrix and the corresponding collagen-modified PVA composite matrix with open-cell foam-wall microarchitectures were studied for evaluation of potential tissue engineering applications. TGA, DTG, DSC, SEM and FTIR results of new bioinspired PVA matrix were employed to build up the effective system identification approach for biomimetic structure, stability, purity, and safety of target soft matrix. The bioinspired PVA matrix and the corresponding collagen-modified PVA composite matrix would be conductive to human hepatoblastoma HepG2 cell proliferation, migration, and expression which might serve as a promising liver cell culture carrier to be used in the biological artificial liver reactor.

## 1. Introduction

For regenerative applications, existing biomedical membranes such as collagen membranes have demonstrated the ability to promote or inhibit cell proliferation [1]. Pure collagens suffer from uncontrollable rapid degradation and weak mechanical strength [2]. Generally, synthetic polymers have better mechanical properties and stability than collagen [3] such as polyvinyl alcohol (PVA), an FDA-approved material widely used in various biomedical applications, including surgical sponges, osteochondral grafts, contact lenses, artificial blood vessels, and implantable medical devices [4,5] (e.g., bone regeneration [6], wound healing [7], and dental applications [8]) because of desirable properties such as biocompatibility, nondegradability, low protein absorption, and easily tunable mechanical properties. PVA matrix was characterized by its super water-absorbent property, great durability and cleaning ability, and its super soft texture when moist. Using sulfuric acid as a catalyst and a suitable pore-forming agent, this porous PVA foam was prepared through PVA acetalization. Usually, some pore-forming agents, such as starch, surfactants or reagents [9,10], could be employed to produce gas during the crosslinking reaction to prepare PVA foam matrix. Additionally, water was used as a pore-forming agent to obtain porous structure without any other additional pore-forming agents [11]. Starch is a pore-forming agent that has been commonly used in the preparation of porous PVA matrix [11]. The average pore diameter of PVA foam varied from 30 to 60 μm when wheat starch was used, and from 60 to 100 μm when potato starch was used [12]. However, the volumes of PVA matrix gradually shrunk with the increasing acetalization degree, and water was continuously excluded during the acetalization process as observation. The PVA matrix with a compacted or closed cell porous structure and a poor interconnectivity was obtained. The resulting PVA foam had a residual pore-forming agent and undesirable degradable products which might be harmful for biomedical applications such as tissue engineering or wound management [13].

The PVA/collagen composite materials were successfully developed in the forms of patches, nanofibers, hydrogels, polymer blend, mixed foam, and dual layers [14,15,16,17,18], which were mainly employed for osteochondral defects applications [14], corneal tissue [15], cartilage tissue engineering [16], and wound healing [17,18]. PVA was employed to modulate the mechanical properties, degradation properties and cell regulation ability of PVA-based composite materials, which would show the ability to regulate the cell adhesion and proliferation behavior of cell and play a critical role in tissue regeneration [19,20]. With a strong focus on therapeutic applications, the characteristics of materials for medical or regenerative applications examine the inspiration from nature [21,22], for example, biomimetic hydrogels for cartilage tissue engineering [23], a biomimetic drug delivery system by integrating grapefruit extracellular vesicles and doxorubicin-loaded heparin-based nanoparticles for glioma therapy [24], and chameleon-inspired multifunctional plasmonic nanoplatforms for biosensing applications [25].

In this study, a novel bioinspired soft matrix with supporting interconnective foam-wall microarchitectures and/or struts made of crosslinked polyvinyl alcohol with air cavities inspired by avian skeleton and feather rachises for drainage medical treatment and regenerative application such as a potential dressing for negative pressure wound therapy [7] or a potential three-dimensional support material [4,5] was designed. The bioinspired soft matrix with high interconnectivity can be fabricated by using the designed air stream pore-forming process. Furthermore, the bioinspired soft matrix was employed to prepare a kind of collagen-containing composite matrix and characterized by Fourier transform infrared spectroscopy (FTIR), thermo-gravimetric analysis (TGA), and scanning electron microscopy (SEM) to obtain the information of identifications, thermal stabilities, microstructures, and interconnectivity.

## 2. Materials and Methods

### 2.1. Materials

Commercial polyvinyl alcohol foam samples PVAM-S1, PVAM-S2, PVAM-S3, and PVAM-S4 were purchased from Kanghua Co., Ltd. (Nantong, China), Yingjia Maidike Co., Ltd. (Beijing, China), Huayang Co., Ltd. (Hengshui, China), and Shandong Weihai Co., Ltd. (Weihai, China), respectively. Bovine Achilles tendon type I collagen was provided from Hebei Collagen Biotechnology Co., Ltd., (Handan, China).

### 2.2. Preparation of Polyvinyl Alcohol Matrix by Using a Biomimetic Airstream Pore-Foaming Process

Polyvinyl alcohol matrix (PVAM) is synthesized from polyvinyl alcohol (PVA) and formaldehyde. A new bioinspired polyvinyl alcohol matrix (PVAM) with fully open cells and channels was designed and prepared by using a designed clean airstream pore-foaming process. The reduced pressure could be employed to build up a clean airstream pore-foaming environment. In a tank, a certain amount of PVA was dissolved in 500 mL deionized water by vigorous stirring with a speed of 10,000 rpm and 0.2 atm at 95 °C until complete dissolution under a clean air environment. The clean airstream was incorporated into the tank for the formation of air cavities bioinspired by avian skeleton and feather rachises with structural pneumaticity. A measure of 90 mL of 24 wt% formaldehyde solution and a certain amount of Triton X-100 were poured into 380 mL of 12 wt% PVA solution under vigorous stirring. The bubble size was regulated by the added amount of Triton X-100. The foam was formed by bubbling air. Liquid froths were obtained after 5 min. Then, 30 mL of 50 wt% sulfuric acid was poured into the above froth at room temperature. After reaching maximum volume, the froths were poured into a mold and cured in a temperature-controlled oven at 60 °C for 8 h and another 8 h under a reduced pressure of 0.2 atm. The resulting samples of porous PVA matrix were washed thoroughly with water at least five times to remove the remaining sulfuric acid and formaldehyde [26].

### 2.3. Preparation of Collagen-Modified Polyvinyl Alcohol Composite Matrix

The PVA matrix was cut into 1.0 cm × 1.0 cm × 0.1 cm rectangular cubes and washed with deionized water. The resulting PVA matrix was soaked in collagen solution of concentration at 0.2% for 12 h to prepare a collagen-modified PVA composite matrix. The resulting collagen-modified PVA composite matrix was washed with deionized water using ultrasonic cleaning method to remove any residual salts and then lyophilized. The collagen-modified polyvinyl alcohol composite matrix (PVACM) was obtained.

### 2.4. Characterization and Cell Morphologies on PVAM and Collagen-Modified PVACM

Chemical transition of the polyvinyl alcohol matrix (PVAM) was analyzed by Fourier-transform infrared spectroscopy (FTIR) (Nicolet iS50, Thermo Fisher, Madison, WI, USA). Transmittance values were recorded in the spectral region from 500 cm^−1^ to 4000 cm^−1^. The thermal degradation behavior of PVAM was recognized as the temperature at the maximum peak. Thermo-gravimetric analysis (TGA) was carried out from room temperature to 500 °C under a nitrogen atmosphere at a heating rate of 20 °C/min. The thermal transition behaviors of the PVAM and starch samples were determined by differential scanning calorimetry (DSC) (Schimadzu DSC-50 (Kyoto, Japan) from 30 °C to 200 °C at a heating rate of 10 °C/min under a nitrogen atmosphere at a flow rate of 30 mL/min. Scanning electron microscopy (SEM) (JSM6700F, JEOL Ltd., Tokyo, Japan) was employed to study the morphologies of HepG2 cells on PVAM and collagen-modified PVACM after three days of cell seeding.

## 3. Results

### 3.1. Morphological Evaluations of Polyvinyl Alcohol Matrix

**Figure 1:**
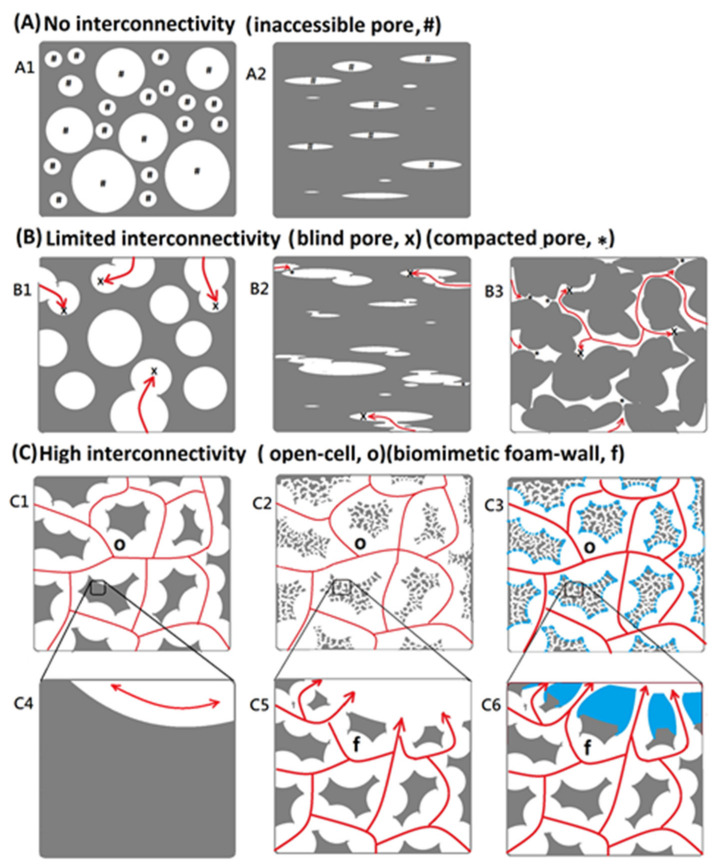
Schematic drawing of (**A**) no interconnectivity (Two types: A1 and A2), (**B**) limited interconnectivity (Three types: B1, B2, and B3), and (**C**) high interconnectivity (Six types: C1, C2, C3, C4, C5, and C6) (“#“: accessible pore; “x”: blindpore; “✽”: compacted pore; “o”: open-cell; and “f”: biomimetic foam-wall).

Porous matrices served as a guide for tissue regeneration as a three-dimensional substrate with temporary mechanical support for cell attachment, proliferation, and infiltration [27]. The morphology is the key feature that affects both the biological and mechanical efficiency of the medical matrices, which would be needed to provide a porous architecture with high interconnectivity to enable cell infiltration, nutrient flow, and integration of the material within the host tissue [28]. Various matrix manufacturing techniques such as emulsion templating [28], gas foaming of heterogeneous blends [29], electrospinning [30], and additive manufacturing [31] had been widely used to introduce porosity into tissue engineering scaffolds. However, PVA matrix always exhibit no interconnectivity or limited interconnectivity because of inaccessible pore, blind pore, and compacted pore within the microstructure, as shown in Figure 1. In this study, a biomimetic airstream pore-foaming process was employed as a new matrix fabrication technique to prepare a bioinspired polyvinyl alcohol matrix (PVAM) with a foam-wall microstructure, which exhibited high porosity and high interconnectivity. The resulting high porosity and interconnectivity enable cell migration, vascularization, and provide space for newly forming tissues to meet the requirements of specific tissue engineering applications and clinic treatments [31,32].

**Figure 2:**
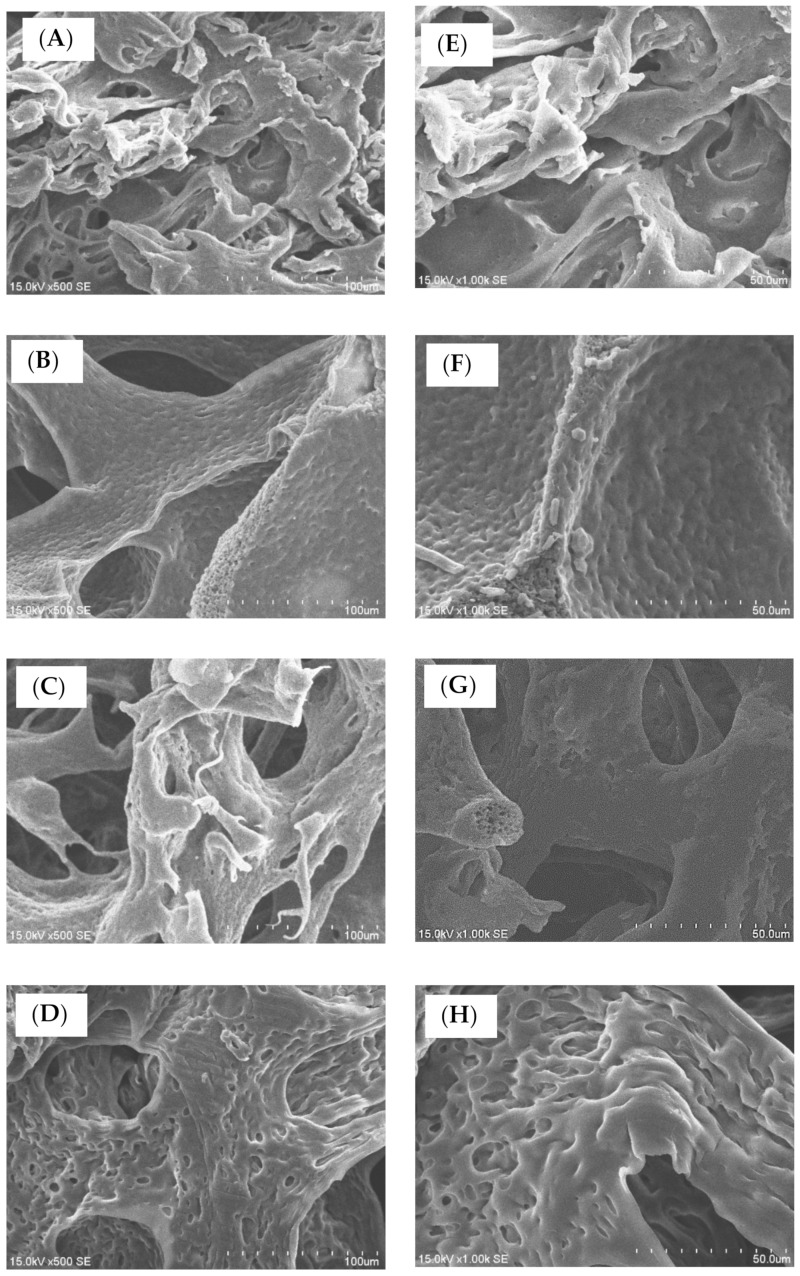
The microstructural morphological evaluations of commercial design of soft medical PVA matrix by using SEM. (**A**) PVAM-S1 (scale bar 100 μm), (**B**) PVAM-S2 (scale bar 100 μm), (**C**) PVAM-S3 (scale bar 100 μm), (**D**) PVAM-S4 (scale bar 100 μm), (**E**) PVAM-S1 (scale bar 50 μm), (**F**) PVAM-S2 (scale bar 50 μm), (**G**) PVAM-S3 (scale bar 50 μm), and (**H**) PVAM-S4 (scale bar 50 μm).

For potential tissue engineering application, a good support and interconnective microstructure was important. Until now, most of commercial medical PVA matrix (such as PVAM-S1, PVAM-S2, PVAM-S3, PVAM-S4) have shown compacted closed-cell microstructures and could not provide a good support and interconnective microstructure, which play an important role as a scaffold for tissue engineering (Figure 2). In general, the compacted closed-cell microstructures might be due to poor foaming ability by using the traditional starch pore-foaming process, air-assisted traditional starch pore-foaming process, or incomplete cross-linking reaction of PVA.

### 3.2. Thermal Stability Evaluations of Medical PVA Matrix

**Figure 3:**
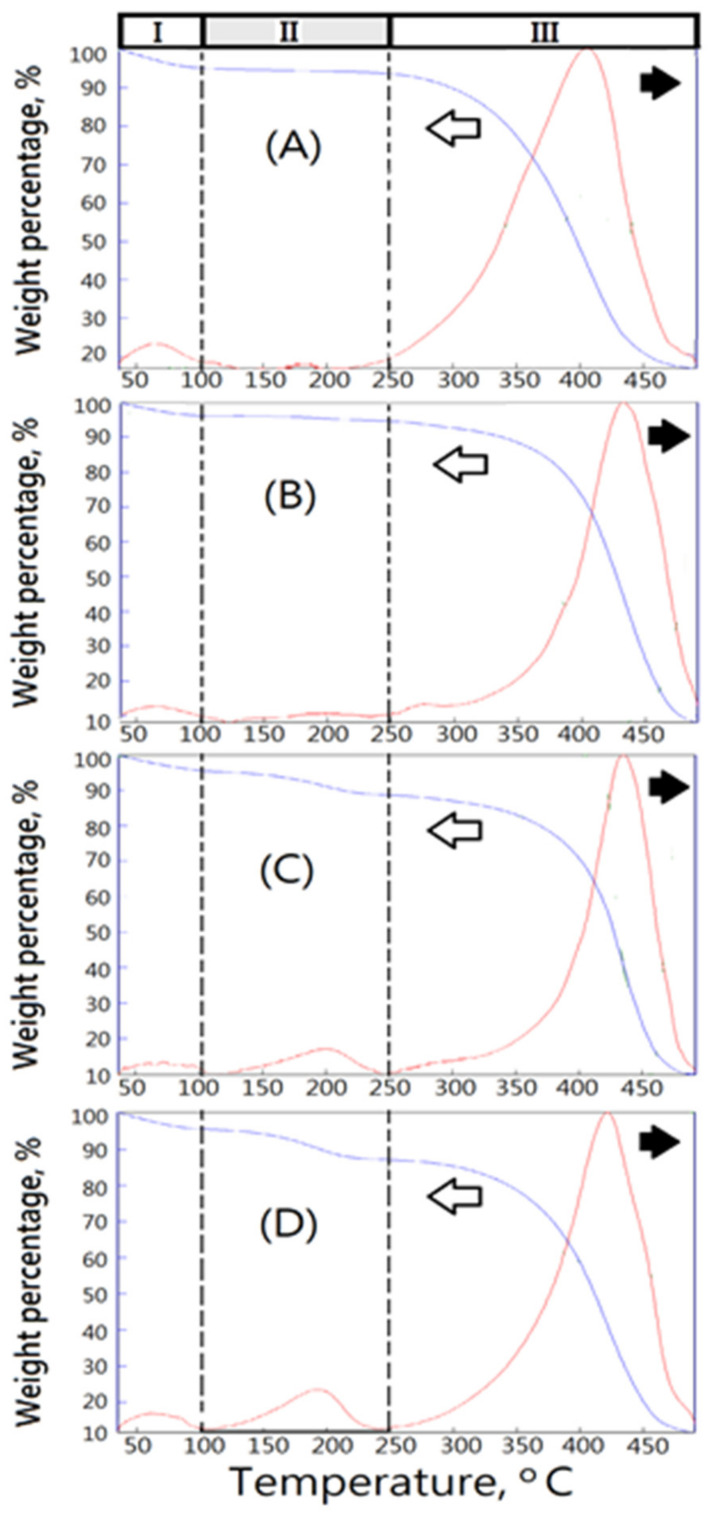
The thermal degradation behaviors of medical PVA matrix by using TGA. (**A**) PVAM-S1, (**B**) PVAM-S2, (**C**) PVAM-S3, and (**D**) PVAM-S4.

To understand the thermal characteristics of the matrix having compacted closed-cell microstructures, the commercial medical PVA matrices, such as PVAM-S1, PVAM-S2, PVAM-S3, and PVAM-S4, were identified by using TGA and DSC. The medical PVA matrix with compacted and closed-cell microstructures exhibited poor thermal stability [33]. With increasing temperature, several thermally degraded products and the weight loss of materials was observed. From Figure 3, the TGA and DTG results of commercial medical PVA matrix showed three stages of weight loss, such as stage I in 50~100 °C, stage II in 100~250 °C, and stage III in 250~500 °C.

First, the weight loss of the commercial medical PVA matrix in stage I at around 100 °C contributed to the loss of water molecules trapped in the hydrophilic PVA. The weight losses of stage I were determined to be 5 wt%, 3 wt%, 4 wt%, and 4 wt% for PVAM-S1, PVAM-S2, PVAM-S3, and PVAM-S4, respectively. Second, TGA and DTG spectra of commercial medical PVA matrix in stage II exhibited slight thermal hydrolysis signals. However, the slight thermal hydrolysis signals exhibited the materials with a poor thermal stable structure, increasing with temperature. T_dmax_,II values could be observed at 180 °C, 200 °C, 200 °C, and 190 °C for PVAM-S1, PVAM-S2, PVAM-S3, and PVAM-S4, respectively. The weight losses of stage II were determined to be about 2 wt%, 1 wt%, 8 wt%, and 9 wt% for PVAM-S1, PVAM-S2, PVAM-S3, and PVAM-S4, respectively. The relative very low values of the initial hydrolysis temperature in stage I were observed at 160 °C, 120 °C, 110 °C, and 110 °C for PVAM-S1, PVAM-S2, PVAM-S3, and PVAM-S4, respectively, which indicated the thermal degradation of the commercial medical PVA matrix would happen easily and caused serious harm for biomedical applications such as biocompatibility and tissue engineering [34].

In the second stage (stage II), the initial thermal degradation temperatures of PVAM-S1, PVAM-S2, PVAM-S3, and PVAM-S4 were very low, at 160 °C, 120 °C, 110 °C, and 110 °C, respectively, indicating that commercial medical drainage PVAF materials were prone to thermal degradation, causing serious harm to biomedical applications such as biocompatibility and tissue engineering. Third, TGA and DTG spectra of commercial medical PVA soft matrix in stage III exhibited a relatively high T_dmax_ value at 405 °C, 435 °C, 435 °C, and 420 °C for PVAM-S1, PVAM-S2, PVAM-S3, and PVAM-S4, respectively. However, all commercial medical PVA matrices showed a broad peak of DTG curve in stage III and the relative low values of initial hydrolysis temperature in stage III were observed at 220 °C, 250 °C, 250 °C, and 250 °C for PVAM-S1, PVAM-S2, PVAM-S3, and PVAM-S4, respectively, which indicated poor thermal structural stability and an incomplete cross-linked structure. For PVAM-S1, the peak of DTG curve in stage III was quite broad, observed in Figure 3A and an incomplete entanglement microstructure with closed cells was observed in Figure 2A.

**Figure 4:**
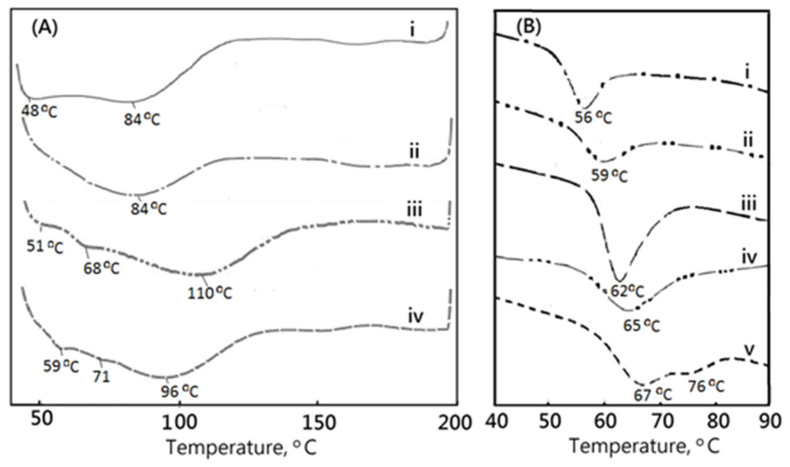
The thermal transition behaviors of (**A**) medical PVA matrix in this work (wherein PVAM-S1 i, PVAM-S2 ii, PVAM-S3 iii, and PVAM-S4 iv) and (**B**) starch materials (wherein wheat starch i, pea starch ii, potato starch iii, cassava starch iv, and rice starch v) by using DSC.

Differential scanning calorimeter analysis (DSC) was also employed for thermal stability evaluation of the commercial medical PVA matrix, such as PVAM-S1, PVAM-S2, PVAM-S3, and PVAM-S4. From Figure 4A, the DSC spectrum of the commercial medical PVA matrix exhibited a peak below 100 °C. It might be due to the residual compounds such as unreacted PVA or starch pore-forming agents. In general, the conventional method of manufacturing a PVA matrix is by using pore-forming agents such as wheat or potato starches [35]. Furthermore, some DSC results of various starches such as wheat starch (i), pea starch (ii), potato starch (iii), cassava starch (iv), and rice starch (v) were employed for comparison with those of the commercial medical PVA matrix, as shown in Figure 4B and Table 1 [36,37,38]. The similar DSC values compared with the results of commercial medical PVA matrix were observed in arrange of 56~76 °C, which contributed to the melting temperature of starch. The DSC values of the commercial medical PVA matrix observed at 84~110 °C might contribute to the amylopectin part in starch. In the case of the amylopectin part in sago starch, the melting temperature peak was between 50 and 150 °C [39]. Most of the commercial products with a compacted and closed-cell microstructure were determined and characterized; the results might contribute to the evident of residual starch after the starch pore-foaming process, which could not provide a good support and drainage microstructure. Moreover, the addition of starch would be harmful to the clinical applications and risks of pollution in the storage system of the resulting soft medical drainage material. The residual starch would degrade with an increasing temperature and enhance risks of treatments.

### 3.3. The Fully Open-Cell Microstructures with Air Cavities, Foam Walls, and Structural Pneumaticity Bioinspired by Avian Rachises

**Figure 5:**
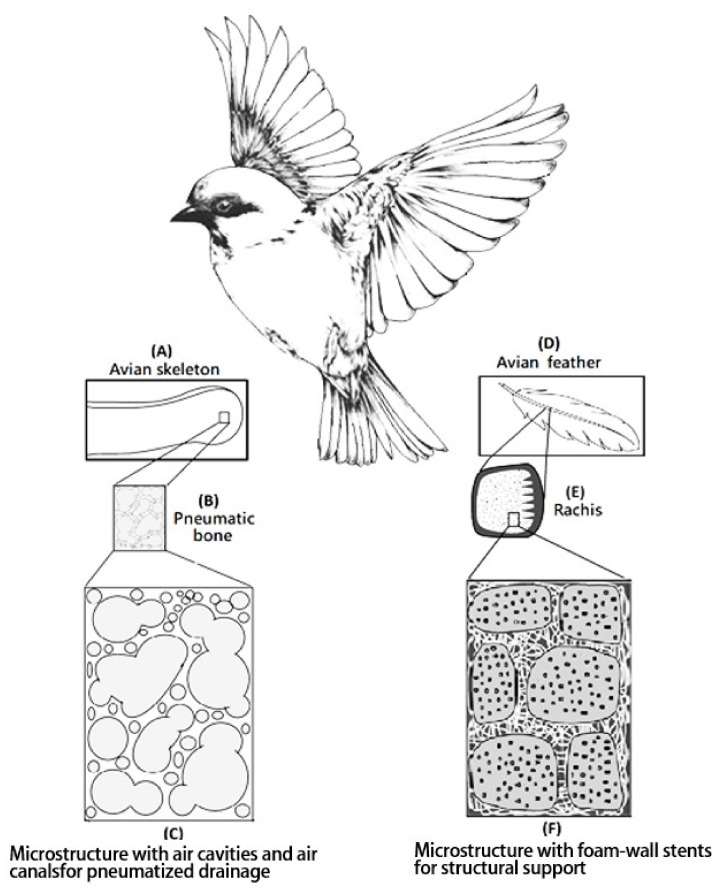
Microstructures were inspired by avian skeleton and feather rachises containing foam-walls, where in (**A**) avian skeleton, (**B**) pneumatic bone, (**C**) microstructure with air cavities and air canals for pneumatized drainage, (**D**) avian feather, (**E**) rachis and (**F**) microstructure with foam-wall strut for structural support.

Biomimetic designs would bring effective materials that are sources of inspiration to biomedical engineers. The fully open-cell microstructures with air cavities, structural foam walls, and structural pneumaticity bioinspired by avian feather rachises and pneumatic bone could be designed. The foam wall showed a “foam-in-a-foam” microstructure and provided internal reinforcements and pneumaticity as shown in Figure 5 [40,41]. Additionally, the corresponding foaming process must be established.

**Figure 6:**
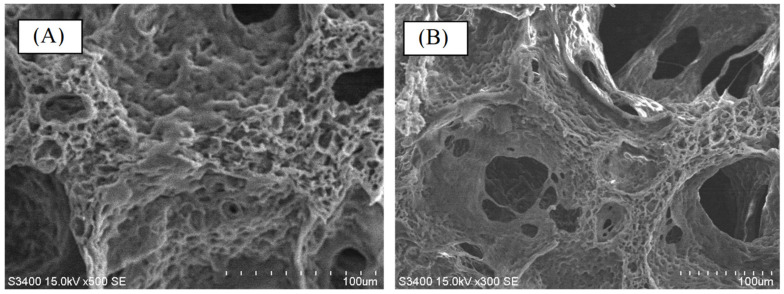
The morphological evaluation of new biomimetic design of bioinspired PVA matrix by using scanning electron microscopy (SEM). (**A**) 500× magnification and (**B**) 300× magnification.

The traditional designs of the polyvinyl alcohol matrix were prepared by traditional starch pore-foaming process or air-assisted starch pore-foaming process. The medical drainage materials with a fully open-cell microstructure could not be obtained. It is difficult to form air cavities to provide interconnectivity and structural support. To build up the air cavities with structural support, foam walls, and structural pneumaticity inspired by avian skeleton and feather rachises, the introduction of clean atmospheric flow in the pore-foaming process of polyvinyl alcohol matrix was possible. Complete crosslinking reaction for preparation of polyvinyl alcohol foam materials was also important. During the starch-containing pore-foaming process, the pore-foaming agent, starch, could not provide enough driving force to form atmospheric flow, which would promote the formation of air cavities with structural support, foam walls, and structural pneumaticity. A new biomimetic design of PVA matrix with air cavities inspired by avian skeleton and feather rachises was prepared by using a designed air flow pore-foaming process without starch pore-foaming agent. A novel PVA matrix (PVAM) with fully open cells and channels could be designed and prepared by using a clean airstream pore-foaming process. The reduced pressure could be employed to build up a clean airstream pore-foaming environment. Furthermore, a morphological evaluation of the result was carried out by using SEM, as shown in Figure 6. The resulting PVA matrix exhibited spongy structure with fully open-cell interconnecting porous network. A highly irregular and deformed porous structure with 100~200 μm pore diameter was shown in Figure 6A. The foam walls with 2~10 μm pore diameter and windows with 20~80 μm pore diameter were observed in Figure 6B.

In this study, the fully open-cell microstructures with air cavities, foam walls, and structural pneumaticity bioinspired by avian rachises could enhance the draining efficiency and structural supporting strength (Figure 5). To build up the stable air cavities, foam walls, and structural pneumaticity bioinspired by avian feather rachises, the introduction of clean atmospheric flow in the foaming process of polyvinyl alcohol matrix would be considered. The clean air was incorporated during the pore-foaming process and crosslinking process for the formation of air cavities, foam walls, and structural pneumaticity bioinspired by avian feather rachises and pneumatic bone, which could be considered as a biomimetic airstream pore-foaming process. The clean air current was employed to avoid impurity.

### 3.4. Thermal Evaluations of New Bioinspired PVA Matrix

In this study, a novel bioinspired polyvinyl alcohol matrix (PVAM) with high interconnectivity for clinic drainage treatment or tissue engineering was designed and prepared. Suitable thermal evaluations of the resulting biomimetic design of soft matrix (PVAM) must be carried out for the identification of thermal structural stability by using TGA, DTG, and DSC.

**Figure 7:**
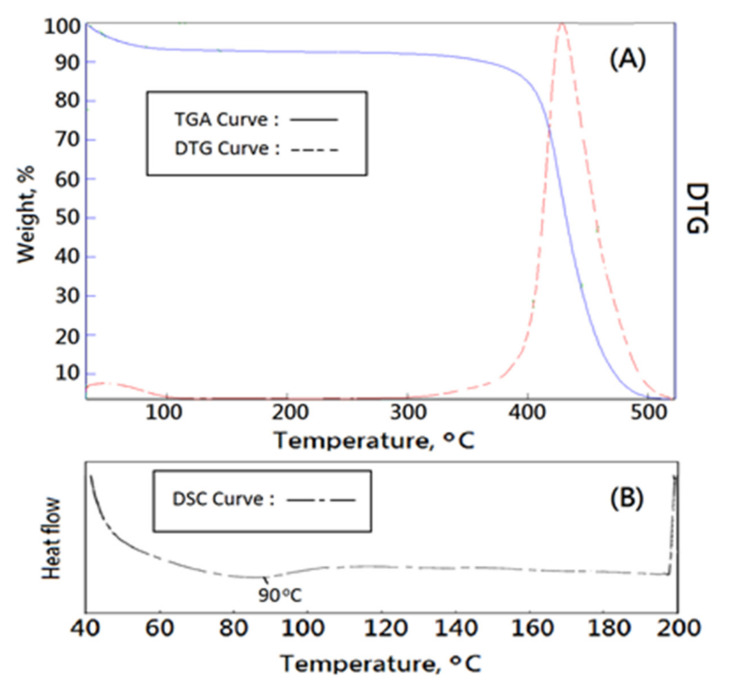
Thermal characteristics of new bioinspired PVA matrix, (**A**) TGA and (**B**) DSC.

From Figure 7, weight loss curves obtained from thermogravimetric analysis (TGA) of biomimetic PVAF could provide some information of thermal degradation such as small molecules, solvent, water, residual reagents, residual foaming agents, starch, weak structural molecules, and prepolymeric molecules, to identify the thermal structural stability. First, TGA and DTG curves of bioinspired PVA matrix in the region with the temperature lower than 100 °C exhibited 6 wt% of weight loss, which would be due to only water molecules escaping from the materials and a good water-absorption property. Second, TGA and DTG curves of bioinspired PVA matrix in the region with a temperature range between 100 and 300 °C exhibited no change of thermal signals. Third, TGA and DTG curves of bioinspired PVA matrix in the region with the temperature higher than 300 °C exhibited a high T_dmax_ value > 425 °C and a narrow peak of DTG curve which indicated high thermal and structural stability and a fully crosslinked structure (Figure 7A). DSC curve of bioinspired PVA matrix was employed to detect traces of pore agents and exhibited a smooth signal below 100 °C, which contributed to escaping water molecules, as shown in Figure 7B.

### 3.5. Fourier-Transform Infrared Spectra of the New Biomimetic Design of Bioinspired Polyvinyl Alcohol Matrix

**Figure 8:**
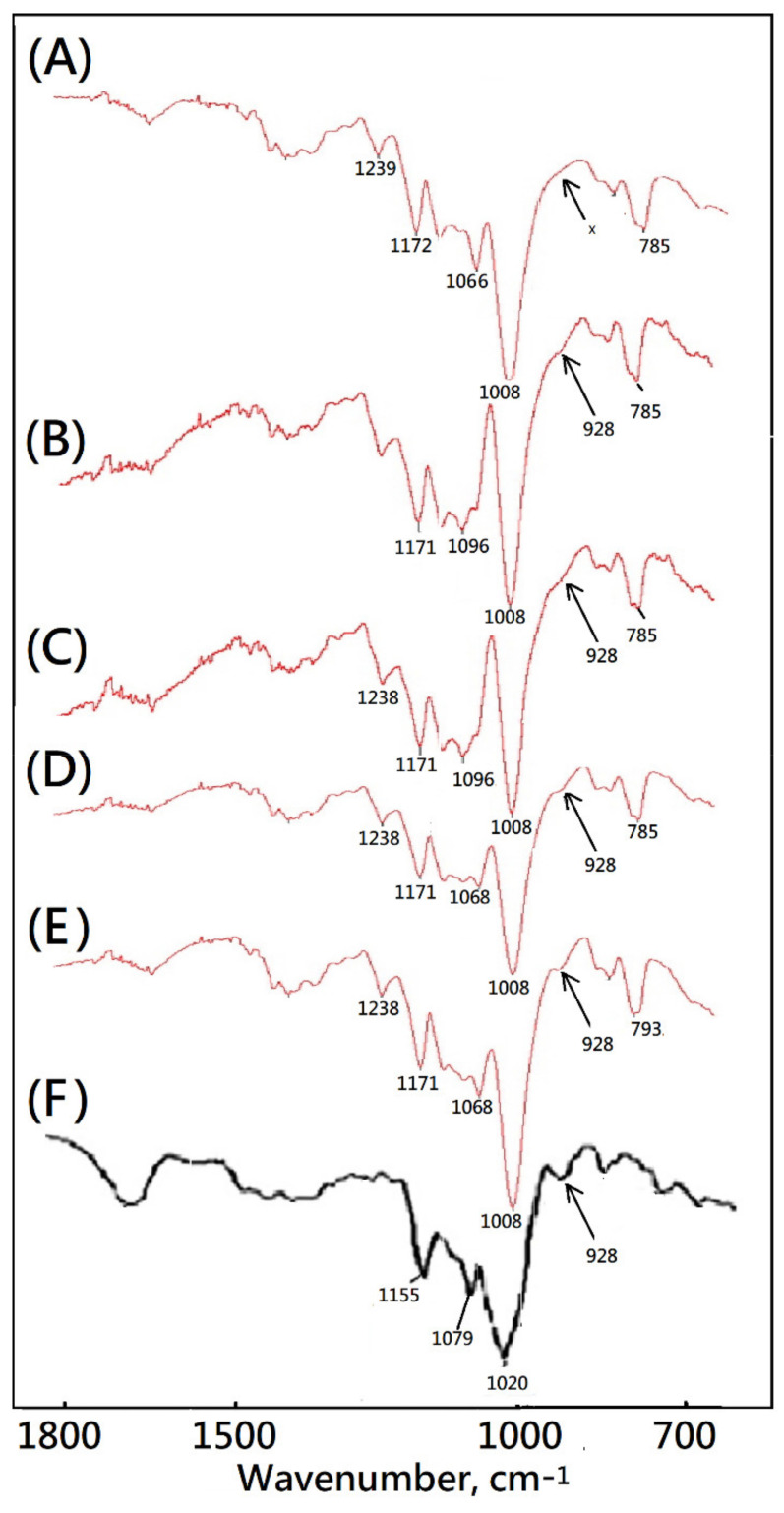
Fourier-transform infrared spectra of medical drainage materials and starch. (**A**) bioinspired PVA matrix, (**B**) PVAM-S1, (**C**) PVAM-S2, (**D**) PVAM-S3, (**E**) PVAM-S4, and (**F**) starch (“x”: no peak).

In this study, a novel bioinspired soft matrix derived from PVA was designed and prepared. The main bands of pure PVA at 3500–3400, 2917, 1425, 1324, and 839 cm^−1^ were assigned to the O–H stretching vibration of the hydroxy group, CH_2_ asymmetric stretching vibration, C–H bending vibration of CH_2_, C–H deformation vibration, and C–C stretching vibration by using FTIR. After the biomimetic airstream pore-foaming process, the molecular structure of PVA matrix was also characterized. The bands at 3500–3400 cm^−1^ were weakened and shifted towards higher frequencies due to the consumption of –OH (due to condensation reaction be acetalization tween PVA and formaldehyde) and the corresponding cleavage of the intra- and intermolecular hydrogen bonding (Figure 8). From Figure 8, the new bands at 2952, 2913, 2861, and 2675 cm^−1^ ascribed to symmetric stretching vibrations of the alkyl CH_2_ group, the new bands at 1239, 1172, 1129, and 1065 cm^−1^ ascribed to the stretching vibration of C–O in C–O–H groups, and a new peak at 1008 cm^−1^ ascribed to –C–O–C–O–C– stretching vibrations confirmed the formation of a formal structure [42,43].

Starch of corn, cassava, and potato showed similar results of absorption bands below 1200 cm^−1^ [44,45]. An absorbance band around 1150 cm^−1^, bands at 1080 and 1020 cm^−1^, and bands around 700–900 cm^−1^ contributed to vibrations of the glucosidic C–O–C bond [43], the anhydroglucose ring O–C stretch [44], and C–O–C ring vibration [43], respectively [43,44]. The absorbance band at 924 cm^−1^ was assigned to vibrational modes of a skeletal glycoside bonds of starches [44]. The FTIR spectrum of the commercial medical PVA matrix showed a similar absorbance band at 924 cm^−1^ as shown in Figure 8. However, the absorbance band at 924 cm^−1^ could not be observed in the FTIR spectra of the new bioinspired PVA matrix. The absorbance band at 924 cm^−1^ might be important evidence for the starch pore-foaming process and an evaluation for a structural support cell microstructure. The fingerprint region at wavenumbers 1200 to 600 cm^−1^ could be employed to identify the purity and molecular structure of suitable medical soft matrix. That is, the fingerprint region of new biomimetic design of medical soft matrix could identify the biomimetic super-clean airstream pore-foaming process different from those of traditional starch pore-foaming process. The similar results were also observed in the results of SEM, TGA, DTG, and DSC as mentioned above. The residual starch would be degraded easily with an increasing temperature. The residual starch pore-foaming agents might be harmful to the cleanliness of the medical soft matrix. The residual starch might enhance risks of pollution in biomedical application such as tissue engineering or wound management [46,47].

**Figure 9:**
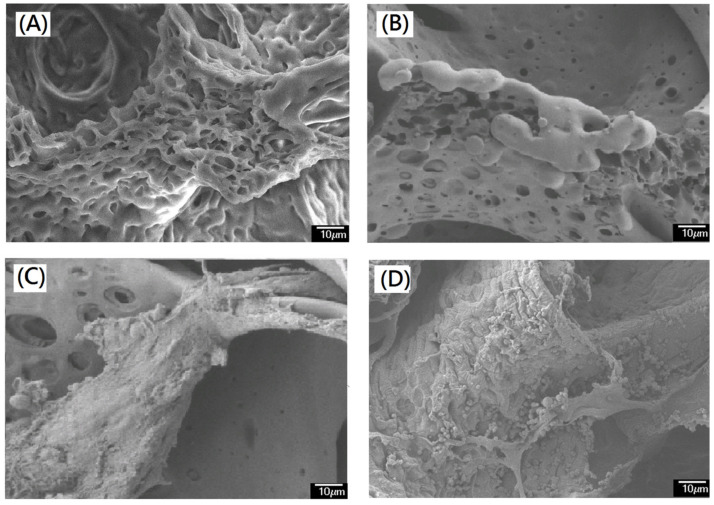
SEM photographs of (**A**) bioinspired polyvinyl alcohol matrix, (**B**) HepG2 cells grew on the bioinspired polyvinyl alcohol matrix after 24 h, (**C**) HepG2 cells grew on the bioinspired polyvinyl alcohol matrix after 48 h, and (**D**) HepG2 cells grew on the bioinspired polyvinyl alcohol matrix after 72 h (scale bar 10 μm).

The ability of cells to adhere and proliferate on the bioinspired polyvinyl alcohol matrix was an important indicator for evaluating the in vivo application potential. At this point, experiments were performed to investigate whether the structure and properties of the matrix scaffold were suitable for cell expansion. The SEM images were used to evaluate the cell morphology on the bioinspired polyvinyl alcohol matrix after various time periods of human hepatoblastoma HepG2 cell culture such as 24 h, 48 h, and 72 h, as shown in Figure 9. After 48 h of culturing, HepG2 cells adhered to the bioinspired polyvinyl alcohol matrix and gradually grew toward the inside of the pores from the interface of the foam wall. After 72 h of culturing, HepG2 cells adhered to the scaffold and grew cover on the interface and inside of the open-cell pores. The foam walls with 2~10 μm pore diameter might provide a rough surface for HepG2 cell proliferation, as shown in Figure 9A,B. The interconnected microporous microstructure of the matrix might well support the penetration of HepG2 cells.

### 3.6. Cell Morphological Observation of a Designed Collagen-Modified Bioinspired Polyvinyl Alcohol Matrix with Open-Cell Foam-Wall Microarchitectures and High Interconnectivity for Potential Tissue Engineering Applications

**Figure 10:**
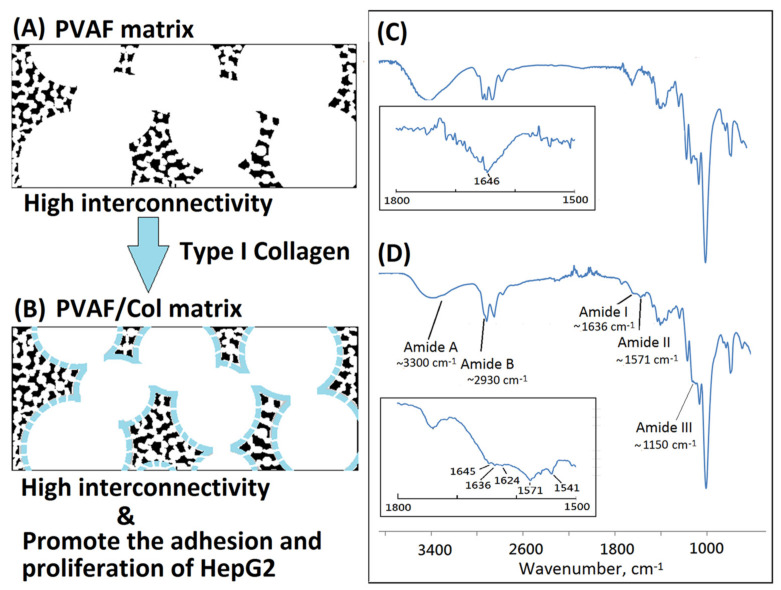
Schematic drawings of the original bioinspired PVA matrix (**A**) and the bioinspired collagen-modified PVA composite matrix (**B**) and FTIR spectra of the original bioinspired PVA matrix (**C**) and the bioinspired collagen-modified PVA (**D**).

**Figure 11:**
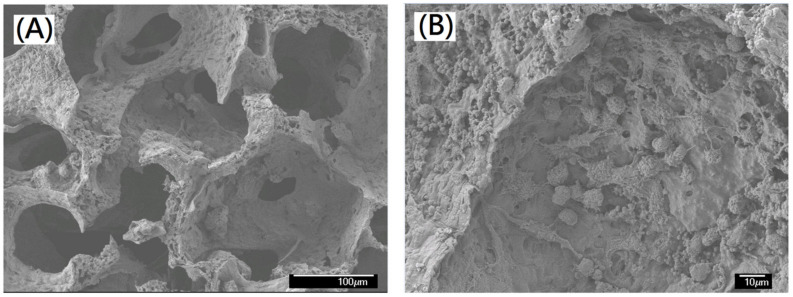
SEM photographs of (**A**) the collagen-modified PVA composite matrix, (**B**) HepG2 cells grew on the collagen-modified PVA composite matrix after 72 h (scale bar 10 μm).

The fabrication of porous matrices as potential hepatocyte carriers for bioartificial liver support have been widely explored [48]. Previous studies have tried to immobilize protein with biomaterials by using covalent binding for HepG2 cell culture [49]. The three-dimensional polyvinyl alcohol matrix may act as a feasible material for artificial liver devices because of biocompatibility, pore-foaming, and mechanical property. However, most of commercial PVA matrices such as PVAM-S1, PVAM-S2, PVAM-S3, or PVAM-S4 were not good enough in both the structural support and interconnectivity to be applied in tissue engineering as a scaffold. In this study, the bioinspired PVA matrix showed a microstructure containing foam walls with good structural support and interconnectivity. Furthermore, the resulting PVA matrix and bovine Achilles tendon type I collagen could be employed to prepare a bioinspired collagen-modified PVA composite matrix with open-cell foam-wall microarchitectures as shown in Figure 9. The hydrophilic hydroxyl group within PVA molecules might provide good binding with the coated bovine Achilles tendon type I collagen molecules by using hydrogen bonding and Van der Waals forces [50,51]. Similar behavior was observed by Zhou et al. [52]. The interaction between PVA and bovine Achilles tendon type I collagen molecules within collagen-modified PVA composite matrix was based on non-covalent interactions such as hydrogen bonding and Van der Waals forces. The hydrogen bonding would be the main force between PVA and collagen interactions, in which the collagen molecule might act as a hydrogen donor and form hydrogen bonds with the hydroxyl group of PVA [53,54]. The bioinspired soft matrix combined the structural properties of the synthetic PVA matrix with the high cell affinity of the type I collagen, thus enhancing the matrix cytocompatibility. Collagen was the dominant component of the extracellular matrix and has been widely used in tissue engineering due to its low antigenicity, good biocompatibility, biodegradability, and non-toxicity [55]. The FTIR analysis was employed to confirm changes of molecular structure after the surface modification on the PVA matrix with collagen molecules. The absorbances at ~3300, ~2930, ~1636, ~1571, and ~1150 cm^−1^ were observed and characterized for amide A (N–H stretching), amide B (the asymmetrical stretching of CH_2_ vibration), amide I (hydrogen bonding between N–H stretching and C=O), amide II (N–H bending and C–N stretching), and amide III (C–N bending, and N–H stretching), respectively, as shown in Figure 10. Remarkably, the resulting bioinspired collagen-modified PVA composite matrix showed quite different absorbance from original bioinspired PVA matrix in the region of 1200~1800 cm^−1^. After the surface modification on the PVA matrix with collagen molecules, the resulting bioinspired collagen-modified PVA composite matrix still sustained a favorable biomimetic interconnected porous structure as shown in Figure 11A.

For the therapeutic application of liver, a bioartificial liver support system has been proposed to support the regeneration of the patient’s liver [56,57,58]. The PVA matrix and biological modification of the PVA matrix might be considered as a suitable material. The human hepatoblastoma HepG2 cells could be employed for in vitro evaluation. The PVA matrix and biological modification of the PVA matrix were a non-toxic material with favorable mechanical properties, chemical stability, and good cell adhesion, which could be easily processed and has a special three-dimensional porous structure [59,60,61]. In this study, the bioinspired PVA matrix was employed to form a relative biocompatible surface of the resulting bioinspired collagen-modified PVA composite matrix which might be suitable for cell adhesion and growth. Some domains in collagen molecules, such as Phe-Hyp-Gly segment, could act as ligands for the integrin family receptors of the cell surface and potentially activate specific biological signals to promote cell attachment and proliferation. The fully open cell and open foam-wall microstructure was maintained after the preparation of a bioinspired collagen-modified PVA composite matrix, as shown in Figure 10A. The morphology of the resulting bioinspired collagen-modified PVA composite matrix with high interconnectivity was also investigated. After 72 h of culturing, HepG2 cells remarkably adhered to the resulting bioinspired collagen-modified PVA composite matrix and grew cover on the interface and inside of the open-cell porous microstructure. The SEM images intuitively displayed the growth and distribution of cells on the resulting bioinspired and collagen-modified PVA composite matrix. HepG2 cells adhering to the type I collagen-modified PVA composite matrix showed filopodia, which indicated that human hepatoblastoma HepG2 cells on type I collagen-modified PVAF matrix composite scaffold in a relatively better proliferative condition than pure PVAF matrix scaffold. The stable adhesion and complete spreading could be observed in Figure 10B. Majhy et al. reported that facile surface modification of substrate could enhance the cell–substrate interaction from a non-adherent state for promoting cell culture [62]. The HepG2 cells attached and grew in a cluster on the bioinspired and collagen-modified PVA composite matrix, which was quite different from the original bioinspired PVA matrix. The bioinspired and collagen-modified PVA composite matrix was conductive to HepG2 proliferation, migration, expression and functionality maintenance. In this work, HepG2 cells proliferated actively and formed cell clusters more efficiently in the collagen-modified PVA composite matrix than original PVA matrix. The collagen of the collagen-modified PVA composite matrix appeared to promote the growth and differentiation of the HepG2 cells. Similarly, Moscato et al. reported that poly(vinyl alcohol)/gelatin hydrogels were good for the growth of HepG2 cells [63]. Furthermore, the morphology of HepG2 cultured on the resulting bioinspired and collagen-modified PVA composite matrix with open-cell foam-wall microarchitectures and high interconnectivity was investigated by SEM. As shown in Figure 11, after 72 h, cells attached and grew well on the collagen-modified PVA composite matrix, and a large number of pseudopods could be seen. The cells which grew upon the collagen-modified PVA composite matrix were significantly higher in quantity than those seen among the PVA. The HepG2 cells would be evenly distributed on the surface of the pore of the PVA composite matrix. In the future, the bioinspired and collagen-modified PVA composite matrix might serve as a promising liver cell culture carrier to be used in the biological artificial liver reactor. Both the bioinspired PVA matrix and the corresponding type I collagen-modified PVA composite matrix could be considered as good materials for different tissue-engineering applications.

## 4. Conclusions

The bioinspired PVA matrix was designed, prepared, and showed relative higher interconnectivity than commercial medical soft matrix, which could be considered as a good potential scaffold for tissue engineering. Furthermore, the bioinspired PVA matrix was employed to prepare a high biocompatible collagen-modified PVA composite matrix after surface modification with type I collagen. In the future, the collagen-modified PVA composite matrix might serve as a promising liver cell culture carrier to be employed in the biological artificial liver reactor.

## Figures and Tables

**Table 1:** Thermal characteristics of medical PVA matrix and starch.

	To ^(a)^, °C	Tp ^(a)^, °C	Tc ^(a)^, °C
PVAM-S1	45 ^(b)^	75 ^(b)^	120 ^(b)^
PVAM-S2	40 ^(b)^	75 ^(b)^	120 ^(b)^
PVAM-S3	40 ^(b)^	56, 65, 115 ^(b)^	150 ^(b)^
PVAM-S4	40 ^(b)^	68, 95 ^(b)^	130 ^(b)^
Potato starch	60.8 [36] (58.6 [37]) (66.1 [38]) ^(c)^	66.6 [36] (63.0 [37]) (72.2 [38]) ^(c)^	75.7 [36] (72.2 [37]) (79.5 [38]) ^(c)^
Wheat starch	56.5 [36] (51.5 [37]) (62.3 [38]) ^(c)^	61.8 [36] (56.2 [37]) (68.2 [38]) ^(c)^	70.7 [36] (61.6 [37]) (75.5 [38]) ^(c)^
Rice starch	60.4 [36] (59.7 [37]) ^(c)^	68.3 [36] (67.8, 75.3 [37]) ^(c)^	79.0 [36] (82.6 [37]) ^(c)^
Corn starch	62.9 [36] ^(c)^	71.2, 80.0 [36] ^(c)^	81.8 [36] ^(c)^
Pea starch	53.5 [37] ^(c)^	59.8 [37] ^(c)^	66.9 [37] ^(c)^
Maize starch	71.9 [38] ^(c)^	76.7 [38] ^(c)^	81.7 [38] ^(c)^
Tapioca starch	63.1 [38] ^(c)^	69.9 [38] ^(c)^	85.9 [38] ^(c)^
Cassava starch	55.8 [38] ^(c)^	65.1 [38] ^(c)^	76.4 [38] ^(c)^

(a) Thermal characteristics of medical drainage materials were obtained by using DSC, To: “onset” initial temperature, Tp: peak temperature, Tc: “endset” final temperature. (b) The results were obtained in this work. (c) Reference to the literature [36,37,38].

## Data Availability

The data presented in this study are available on request from the corresponding author.
